# Calcium Channel Blocker-Associated Chyloperitoneum in Patients Receiving Peritoneal Dialysis: A Systematic Review

**DOI:** 10.3390/ijerph16081333

**Published:** 2019-04-13

**Authors:** Seungyeon Kim, Yun Mi Yu, Jeongyoon Kwon, Hyejin Yoo, Sun Hoi Jung, Euni Lee

**Affiliations:** 1 College of Pharmacy & Research Institute of Pharmaceutical Sciences, Seoul National University, Seoul 08826, Korea; gsy92@snu.ac.kr (S.K.); pp77girl@snu.ac.kr (J.K.); hyejin0414@snu.ac.kr (H.Y.); 2 Department of Pharmacy and Yonsei Institute of Pharmaceutical Sciences, College of Pharmacy, Yonsei University, Incheon 21983, Korea; yunmiyu@yonsei.ac.kr; 3 Department of Pharmaceutical Medicine and Regulatory Sciences, Colleges of Medicine and Pharmacy, Yonsei University, Incheon 21983, Korea; 4 Department of Pharmacy, Seoul National University Boramae Medical Center, Seoul 07610, Korea; shoijung@hanmail.net

**Keywords:** calcium channel blockers, chylous ascites, turbid peritoneal dialysate, triglycerides, lercanidipine, manidipine

## Abstract

Unlike chyloperitoneum associated with clinical conditions including cancer, cirrhosis, and traumatic surgery, calcium channel blocker (CCB)-associated chyloperitoneum is rarely discussed in comprehensive studies on chyloperitoneum. We aimed to investigate the prevalence and characteristics of CCB-associated chyloperitoneum in peritoneal dialysis (PD) patients. The MEDLINE, Embase, CENTRAL, CiNii, and RISS databases were systematically searched for clinical studies on CCB-associated chyloperitoneum in PD patients published up to 31 July 2018. A total of 17 studies (four cohort studies, one case series, and 12 case reports) were selected. Eight CCBs, namely amlodipine, benidipine, diltiazem, lercanidipine, manidipine, nifedipine, nisoldipine, and verapamil, were reported to be associated with chyloperitoneum; manidipine and lercanidipine were the most frequently reported. The average prevalence of chyloperitoneum for lercanidipine was 25.97% in three cohort studies, two of which had a moderate or high risk of bias. Most of the studies revealed chyloperitoneum development within 4 days of initiation of CCB therapy and chyloperitoneum disappearance within 24 h of CCB withdrawal. The results of this study emphasise on the need for awareness among healthcare professionals regarding CCB-associated chyloperitoneum in PD patients. Further studies elucidating the causality and clinical implication of CCB-associated chyloperitoneum are needed.

## 1. Introduction

Hypertension is a common comorbidity in end-stage renal disease (ESRD) patients who have undergone dialysis, with reported prevalence ranging between 72% and 88% [1]. Since cardiovascular diseases, including hypertension, are associated with high mortality rates in patients with ESRD [2], achieving target blood pressure is crucial and often challenging in this population. According to two studies analysing the US Renal Data System database, calcium channel blockers (CCBs) are the antihypertensive drugs most commonly prescribed (approximately 70% of the cases) to patients with ESRD [3], and are associated with reduced rate of all-cause and cardiovascular mortality [4].

Chyloperitoneum is a rare condition characterised by milky-appearing dialysate (from peritoneal fluid) containing high triglyceride concentration [5]. Although its prevalence has not been well established, some studies reported the prevalence to be one per 20,000 to 187,000 admissions at large tertiary hospitals [6,7]. Chyloperitoneum was classified into traumatic and non-traumatic by aetiology, which included cancer, cirrhosis, infections (i.e., tuberculosis), congenital disorders, autoimmune diseases, retroperitoneal fibrosis, and radiotherapy [8]. In addition, certain drugs including aliskiren [9] and CCBs has been reported to cause chyloperitoneum until recently. In 1991, Yoshimoto and colleagues described the first two cases of chyloperitoneum following administration of manidipine in patients receiving peritoneal dialysis (PD) [10]. Since then, several cases of CCB-related non-infectious chyloperitoneum (in the form of turbid peritoneal dialysate) have been reported mainly in patients receiving PD [11,12,13].

CCB-associated chyloperitoneum is rarely discussed in the literature on chyloperitoneum and is hardly described in the information on approved drugs from the U.S. Food and Drug Administration (FDA), the European Medicines Agency (EMA), and the Ministry of Food and Drug Safety in South Korea. Because turbid peritoneal dialysate is commonly regarded as a sign of infectious peritonitis, which is a major clinical complication of PD occurring in more than 70% of peritonitis cases, the condition usually leads to a series of diagnostic examinations (e.g., paracentesis, imaging studies) and empirical antibiotic therapy in clinical setting [14]. When CCB-associated non-infectious chyloperitoneum is misinterpreted as infectious, health care burden can be increased potentially by unnecessary lab tests and inappropriate prescriptions of antibiotics.

Since enhanced understanding of the characteristics of CCB-associated chyloperitoneum is needed for healthcare professionals who prescribe CCBs and establish plans for monitoring, a systematic review was prepared with aims to describe the type of CCBs, prevalence, and timeline of CCB-associated chyloperitoneum in PD patients.

## 2. Methods

### 2.1. Search Strategy

A systematic review was conducted in accordance with the Preferred Reporting Items for Systematic Reviews and Meta-Analyses (Table S1) [15]. We searched the MEDLINE, Embase, Cochrane Central Register of Controlled Trials (CENTRAL), Citation Information by National Institute of Informatics (CiNii; Japan), and Research Information Sharing Service (RISS; Korea) databases to identify as many CCBs approved in the global market as possible.

Since CCB-associated chyloperitoneum has barely been reported, we tried to find all published studies in the form of controlled trials and observational studies, including even case series and case reports. Review articles, comments and conference abstracts were excluded owing to insufficient information. All eligible studies were identified up to 31 July 2018. No language restrictions were imposed. Additional articles were identified and included by manual review of the bibliography lists of the retrieved articles.

Selection criteria included patients who received PD with a CCB as pharmacotherapy and showed turbid peritoneal dialysate, in particular chyloperitoneum, as an outcome. The following search keywords were used: (i) ‘calcium channel blocker’ OR ‘calcium antagonist’ OR the generic name of individual CCBs; (ii) ‘peritoneal dialysis’; (iii) ‘chyloperitoneum’ OR ‘chylous ascites’ OR ‘turbid peritoneal fluid’ OR ‘cloudy dialysate’; and (iv) ‘adverse drug reaction’ OR ‘safety’ OR ‘toxicity’ (Table S2).

### 2.2. Study Selection

One researcher (S.K.) identified articles according to the search strategy, and a second researcher (Y.M.Y) confirmed the process. After removing duplicate studies, two researchers (S.K. and Y.M.Y.) independently selected studies by reviewing the titles, abstracts, and full text according to the eligibility criteria. Any discrepancy in study selection between the two researchers was resolved by consensus involving participation of a third investigator (E.L.). We included only full-text studies containing sufficient relevant information.

### 2.3. Data Extraction and Analysis

The following data were extracted from the included studies by means of a standardised form: (1) study settings (country, study design); (2) demographics of the study population (number of patients, age, and sex); (3) clinical characteristics of the study population (duration of PD treatment, generic names and dosages of CCBs, and duration of CCB therapy); and (4) outcome features (number of cases or prevalence of chyloperitoneum, clinical features of chyloperitoneum, laboratory test results of dialysate and serum, time to onset of chyloperitoneum, and results of withdrawal and re-administration of CCBs).

To identify the studies directly relevant to CCB-associated chyloperitoneum, any potential cause of chyloperitoneum other than CCB, such as microbial culture testing, computed tomography, or positron emission tomography, was collected if indicated in the studies. In terms of study design, any study that reported the incidence or prevalence of chyloperitoneum after initiation of CCB therapy by retrospective or prospective follow-up were considered a cohort study [16]. The included studies were qualitatively summarised. We analysed the studies descriptively and the average prevalence of chyloperitoneum was calculated by dividing the total number of patients with chyloperitoneum by the total number of patients participating in the included studies.

Quality of the included cohort studies was independently assessed by two researchers using a modified Newcastle–Ottawa Scale (Table S3) [17,18], which included the following seven items grouped into three domains: (1) the selection domain (representativeness, ascertainment of exposure, and starting condition prior to outcome), (2) comparability domain (adjustment of data for confounding factors); and (3) outcome domain (ascertainment of outcome, sufficiency of follow-up period, and adequacy of follow-up). Scores (zero or one point) were assigned to each item according to the predefined criteria, and the total score was calculated. Inconsistencies in quality assessment were resolved by consensus, involving the third investigator. For quality assessment of the included case series and case reports, the two researchers independently assessed the causal relation between chyloperitoneum and the presumed causative drugs in the case reports, using the World Health Organization-Uppsala Monitoring Centre criteria [19] and the Naranjo scale [20]. Causality was subdivided into four categories: certain/definite, probable, possible and unlikely/doubtful [19,20].

For quantitative analysis, individual study estimates for degree of association with chyloperitoneum occurrence were reported as odds ratios (ORs) and standardised mean differences (SMD), with corresponding 95% confidence intervals (CIs), for dichotomous and continuous variables, respectively. The estimates were combined using a random-effects model, and heterogeneity was assessed using the I^2^ statistic. Data analysis was performed using SPSS version 22.0 (SPSS Inc., Chicago, IL, USA) and Comprehensive Meta-Analysis version 2.2.064 (Biostat, Englewood, NJ, USA). The significance level was set at *p* < 0.05.

## 3. Results

### 3.1. Literature Search and Study Characteristics

**Figure 1:**
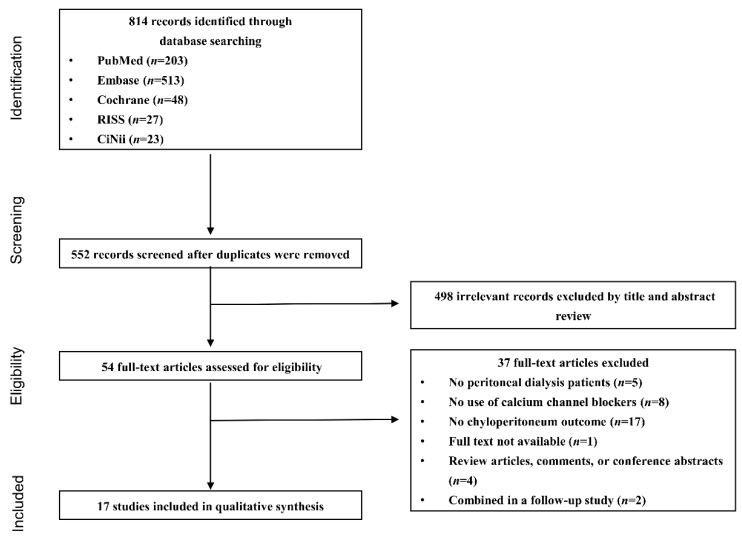
Flow chart of the study selection process.

Among the 552 identified non-duplicate studies, 498 did not meet the inclusion criteria and were excluded after review of the articles’ titles and abstracts. The remaining 54 studies were assessed for eligibility by reviewing the full text, 37 of which were subsequently excluded for the following reasons: (1) the study populations did not receive PD (*n* = 5); (2) the drug intervention did not include a CCB (*n* = 8); (3) chyloperitoneum was not reported as an outcome (*n* = 17); (4) the articles were review articles, comments, or conference abstracts (*n* = 4); (5) the full text of the article was not available even after its corresponding author was contacted by email (*n* = 1); and (6) an earlier study of the same cases were included in a follow-up study (*n* = 2). Finally, 17 studies, including four cohort studies [11,21,22,23], one case series study [24], and 12 single-case reports [12,13,25,26,27,28,29,30,31,32,33,34], were selected and included in our systematic review (Figure 1).

The included studies were conducted in various countries from Asia and Europe, such as Japan (*n* = 5) [11,26,27,28,29], Spain (*n* = 3) [12,25,33], Taiwan (*n* = 3) [21,23,30], India (*n* = 2) [13,31], South Korea (*n* = 1) [29], Italy (*n* = 1) [33], Northern Ireland (*n* = 1) [34], and Turkey (*n* = 1) [22], and published from 1993 to 2018. Four cohort studies were included in this systematic review—three retrospective studies [11,22,23] and one prospective study [21]. There were a total of eight CCBs identified as the cause of chyloperitoneum from this systematic review, including six dihydropyridine CCBs (amlodipine [13,34], benidipine [11], lercanidipine [21,22,23,30,32,33], manidipine [11,12,24,25,26,27,28,29], nifedipine [11], and nisoldipine [11]) and two non-dihydropyridine CCBs (diltiazem [31], and verapamil [24]).

### 3.2. Characteristics of Chyloperitoneum

Among the four cohort studies, three evaluated the effects of the CCB lercanidipine, [21,22,23], whereas one (by Yoshimoto et al.) addressed the effects of nine different CCBs [11]. Out of the nine CCBs (i.e., manidipine, benidipine, nisoldipine, nifedipine, nitrendipine, nilvadipine, nicardipine, barnidipine, and diltiazem), four (i.e., manidipine, benidipine, nisoldipine, and nifedipine) were associated with chyloperitoneum development in patients receiving continuous ambulatory PD, with mean prevalence of 7.57% (19 of 251 patients) [11] (Table 1). The reported prevalence of chyloperitoneum by lercanidipine ranged from 13.04% [22] to 57.14% [23], with an average prevalence of 25.97% (20 of 77 patients). In three cohort studies, turbid peritoneal dialysate developed within four days after initiation of CCB therapy [11,21,23] and disappeared within 24 h after CCB withdrawal, with recurrence of cloudy peritoneal dialysate upon re-administration of the suspected culprit CCBs [11,21,23]. The mean triglyceride concentrations in dialysate in two cohort studies [21,23] were 19.3 and 128.4 mg/dL, respectively.

In the studies by Hsiao et al. [21] and Yang et al. [23], the proportion of male patients was higher in the turbid-dialysate group than in the non-turbid-dialysate group (44.4% vs. 38.7%, 62.5% vs. 33.3%, respectively) and the mean duration of PD treatment was shorter in the turbid-dialysate group than in the non-turbid-dialysate group (33 ± 21 vs. 39 ± 24 months, 20.4 ± 21.6 vs. 33.6 ± 24 months, respectively) (Table 1). In quantitative analysis, however, no significant difference in the incidence of chyloperitoneum caused by lercanidipine was reported between sexes (unadjusted OR 1.72, 95% CI 0.49–5.95, *p* = 0.395) (Figure S1). In addition, there was no significant difference in age (SMD −0.18, 95% CI −1.19–0.84, *p* = 0.730), duration of PD treatment (SMD −0.36, 95% CI −0.97–0.25, *p* = 0.250), and serum triglyceride concentrations (SMD 0.03, 95% CI −1.24–1.30, *p* = 0.964) in patients with chyloperitoneum, compared with those in patients without chyloperitoneum (Figure S2). Low statistical heterogeneity was observed for sex (I^2^ = 0%) and duration of PD treatment (I^2^ = 0%), but high heterogeneity was noted for age (I^2^ = 59.28%) and serum triglyceride concentrations (I^2^ = 72.84%) (Figures S1 and S2).

From the case series and case report studies, we identified 15 adult cases [mean age (SD) = 51.73 years (9.65)] and most of the cases were of males (64.29%), except for one case in which sex was not disclosed. A paediatric case of CCB-associated chyloperitoneum (seven months old, male) was also included (Table 2). Triglyceride concentrations in the dialysate were below 200 mg/dL in 75.0% of chyloperitoneum cases and serum triglyceride concentration ranged from 81 to 182 mg/dL. The mean duration of PD treatment was 6.56±9.86 months in 13 patients. In approximately two-thirds of the total cases [12,13,25,26,27,28,29,30,32,34], cloudy peritoneal dialysate was observed within four days after CCB therapy initiation, and in 11 cases, peritoneal dialysate cleared up within 24 h after CCB withdrawal [12,13,21,22,23,24,25,26]. In five cases [25,30,31,32,34] in which CCB therapy was resumed, peritoneal dialysate clouding reappeared and increased triglycerides recurred.

### 3.3. Study Quality

In quality assessment, one cohort study [23] was found to have a low risk of bias, with a modified Newcastle-Ottawa Scale score of 6 points (Table 1). The remaining three studies [11,21,22], which showed poor representativeness of study population, poor ascertainment of outcome, and insufficient follow-up were revealed to have moderate or high risk of bias, with a score of 3–5 points. Details of the overall quality assessment of the four cohort studies are described in Table 3.

The causality of the identified cases was deemed to be certain/definite in four cases [25,30,31,32], and probable in the other 12 cases owing to a lack of information on CCB re-challenge [12,13,24,26,27,28,29,33] or existence of alternative or possible causes [34] (Table 2).

## 4. Discussion

To the best of our knowledge, this is the first systematic review of the characteristics of drug-related chyloperitoneum in patients receiving PD. Previous reviews have examined the association between chyloperitoneum and clinical conditions, such as cancer, cirrhosis, and traumatic surgery [6,35,36]. One of the key findings of this study is that a specific drug-class, i.e., CCB drugs, was associated with chyloperitoneum, and both CCB subclasses, dihydropyridine and non-dihydropyridine, were linked to this condition. Manidipine and lercanidipine, which are dihydropyridine-type CCBs approved and used in Asian (e.g., South Korea, Japan, and Taiwan) and European countries (e.g., United Kingdom, France, Austria, and Germany), were the most frequently reported CCBs associated with chyloperitoneum.

The proposed mechanisms of chyloperitoneum associated with surgery, malignancy, or cirrhosis which are relatively well-known causes are related with lymphatic functions. Invasion and disruption of lymphatic vessels resulting in abnormal lymph flow or lymph production are suggested to cause chyloperitoneum development [8], and CCB-associated chyloperitoneum is anticipated to have similar mechanism. The mechanism underlying the development of CCB-associated chyloperitoneum presumably involves impairment of lymphatic functions in triglyceride disposal and increased ultrafiltration through the peritoneal membrane [37]. Given that manidipine and lercanidipine are highly lipophilic [38], they can easily penetrate the lipid bilayer of the cell membrane and act on calcium channels in both smooth muscle cells of the gut and lymphatic vessels [21,24]. Although the high lipophilicity of manidipine and lercanidipine may explain, in part, why these two drugs are frequently reported, further research is needed to determine whether other highly lipophilic CCBs, such as barnidipine or lacidipine [39], are associated with chyloperitoneum development.

Our quantitative analysis revealed that characteristics such as age, sex, duration of PD treatment, and serum triglyceride concentrations were not significantly related to lercanidipine-associated chyloperitoneum. However, only two studies provided sufficient detailed information that allowed quantitative analyses by age, sex, triglyceride levels, and duration of PD; hence, a quantitative analysis of these two studies was conducted. In addition, there was high heterogeneity in the effects of age and serum triglyceride concentrations on chyloperitoneum development. The relatively high heterogeneity among the included cohort studies and the small sample sizes may have affected the reliability of our results on the estimated incidence of CCB-associated chyloperitoneum and the effects of predictive factors. Although some studies reported that net ultrafiltration [21,23], dialysate-to-plasma ratio of creatinine [21], and cholesterol concentrations in dialysate [23,27,29,33] increased in the turbid-dialysate group, quantitative analysis could not be conducted because of a lack of detailed information.

Increased triglyceride concentration in dialysate is considered an important indicator of chylous ascites, but its precise diagnostic value remains to be determined. Triglyceride concentrations above 200 mg/dL have been used by some groups as an arbitrary diagnostic criterion for chyloperitoneum [7], as well as 110 mg/dL as the cut-off value [40]. In our study, however, less than 30% of all chyloperitoneum cases had triglyceride concentrations exceeding 200 mg/dL. Two of the included cohort studies also showed mean triglyceride concentrations in dialysate of below 200 mg/dL in the chyloperitoneum group. This finding might be attributed to the fact that triglyceride concentrations of ascites in patients receiving PD were diluted by peritoneal dialysate, which is administrated about 2 L per time and is exchanged four times a day [21]. Additionally, triglyceride concentrations above 65 mg/dL have been proposed as the best discriminatory criterion in a study analysing the value of lipids for distinguishing between cirrhotic and malignant chylous ascites [41]. Although a cut-off triglyceride concentration of 200 mg/dL has been the most conservative measure, the use of this cut-off triglyceride concentrations to assess the presence of chyloperitoneum could lead to misclassification bias.

Our systematic review showed that most of the studies indicated that CCB-associated chyloperitoneum developed within 4 days of initiation of CCB therapy and disappeared within 24 h of CCB withdrawal. In most cases, chyloperitoneum developed in patients who received their first CCB treatment, whereas some studies reported that chyloperitoneum developed after the CCB type prescribed to patients was changed [12] or when the dosage of the same drug was increased [26,42]. These findings indicated the timeline for medication review or monitoring of chyloperitoneum while PD patients are receiving pharmacotherapy. The management and diagnostic pathway of PD patients who develop chyloperitoneum generally include empirical antibiotic therapy and abdominal paracentesis [23] or imaging studies and other invasive tests [7,24,40]. Although these diagnostic processes can be time-consuming, expensive, and potentially harmful to patients, we cannot suggest the suspension of CCBs without an extensive diagnostic workup. However, we believe that awareness of CCB-associated chyloperitoneum in healthcare professionals is needed to be able to suspect a CCB as one of the causes of chyloperitoneum and to consider discontinuing the suspected CCB, if needed, when infection is rule out as the cause of chyloperitoneum.

Our study has some limitations. First, the mean prevalence of lercanidipine-associated chyloperitoneum determined in our study was relatively high (i.e., 25.97%). Although chyloperitoneum is generally known to be an uncommon condition and its incidence and prevalence are still unknown, some authors have found that the prevalence rates of chyloperitoneum due to trauma, a relatively well-known cause, range from 1.1–7.4% [5]. The relatively high prevalence rate reported in our study might be caused by poor quality of the study population in the included observational studies, which offered no explicit description of representativeness of the exposed cohort or selection criteria for participants. Therefore, our result should be interpreted with caution for use in clinical settings. Second, the frequently reported manidipine-associated chyloperitoneum cases from our study should also be considered in the context of the prescribing patterns of each country in selecting the type of CCBs for PD patients. Third, our systematic review, which included case reports and case series, may not be adequate to infer causal association. Although these studies have low validity with respect to their study designs, all cases were indicative of ‘certain’ or ‘probable’ causality. Owing to the inherent limitations of the study design and potential publication bias, more rigorous studies (e.g., analysis of post-marketing surveillance data) indicating a confirmative association between CCBs and chyloperitoneum are needed.

## 5. Conclusions

CCB-associated chyloperitoneum is very uncommon, yet continuously detected, in PD patients. Our study—the first systematic review on CCB-associated chyloperitoneum—indicated that two highly lipophilic CCBs (manidipine and lercanidipine) were the most frequently reported to be associated with chyloperitoneum. No predisposing factors, such as sex, age, duration of PD treatment, and serum triglyceride concentrations, could be drawn from our results. Further research is needed on the risk of CCB-associated chyloperitoneum in PD patients. If necessary, monitoring for chyloperitoneum occurrence should be scheduled at least four days after initiation of CCB therapy in PD patients. Our study also highlighted the need for awareness of CCB-associated chyloperitoneum in PD patients among healthcare professionals.

## Figures and Tables

**Table 1:** Characteristics of the included cohort studies.

Study	Country	Study Design	Number of Patients	Drug Name	Dose (mg)	Prevalence of CP	Age (mean ± SD, year)		Sex (men, %)		TGs in Dialysate (mean ± SD, mg/dL)		TGs in Serum (mean ± SD, mg/dl)		Duration of PD Treatment (mean ± SD, months)		Time-to-Onset of CP (mean ± SD, days)	Result of Withdrawal	Result of Rechallenge	QA Score (0–7) ^a^
							T	Non-T	T	Non-T	T	Non-T	T	Non-T	T	Non-T				
Hsiao 2010 [21]	Taiwan	P	40	Lercanidipine	5	22.5% (9/40)	39.4 ± 14.3	47.5 ± 12.5	44.4	38.7	19.3 ± 6.3	0	123 ± 43	151 ± 52	33 ± 21	39 ± 24	1.2 ± 0.4	Dialysate clear within 24 h	Cloudy again	5
Yang 2008 [23]	Taiwan	R	14	Lercanidipine	-	57.14% (8/14)	52.6 ± 18.5	46.0 ± 10.8	62.5	33.3	128.4	6.5	218.0 ± 176.6	115.8 ± 3.2	20.4 ± 21.6	33.6 ± 24	38.5 ± 60.8	Dialysate clear within 24 h	Cloudy again	6
Topal 2006 ^b^ [22]	Turkey	R	23	Lercanidipine	5	13.04% (3/23)	45.3 ± 17.6		39.1		-		-		15.9±11.6		1	-	-	3
Yoshimoto 1998 [11]	Japan	R	251	Various CCBs ^c^	-	7.57% (19/251)	55 ± 17	50 ± 12	52.6	67.7	-		-		-		-	Dialysate clear	Cloudy again	4

^a^ The quality of the studies was assessed using a modified Newcastle-Ottawa Scale comprising of seven items, each scoring either 1 or zero point (Table S3). ^b^ This study uncovered the following characteristics of the entire study sample: mean age 45.3 years, 39.1% male (*n* = 9) and duration of PD treatment 15.9 months. ^c^ CCBs (number of CCB-treated patients with chyloperitoneum versus total number of CCB-treated patients) included in the study were manidipine (15/36), benidipine (2/2), nisoldipine (1/11), nifedipine (1/159), nitrendipine (0/2), nilvadipine (0/7), nicardipine (0/25), barnidipine (0/1) and diltiazem (0/8). Abbreviation: CCB, calcium channel blocker; CP, chyloperitoneum; PD, peritoneal dialysis; P, prospective; QA, quality assessment; R, retrospective; SD, standard deviation; T, turbid; TGs, triglycerides.

**Table 2:** Characteristics of the included case series and case report studies.

Study	Country	Study Design	Number of Patients	Drug Name	Dose	Age	Sex	TGs in Dialysate (mg/dL)	TGs in Serum (mg/dL)	Duration of PD Treatment	Time-to-Onset of CP	Result of Withdrawal	Result of Rechallenge	Causality ^a^
Betancourt 2013 [24]	Spain	Case series	4	Manidipine	-	60 y	M	52	-	2 mo	-	Dialysate clear	-	Probable
				Verapamil	-	41 y	M	9	-	4 mo	-	Dialysate clear	-	Probable
				Manidipine	-	70 y	F	745	-	4 d	-	Dialysate clear	-	Probable
				Manidipine	-	52 y	M	452	-	5 mo	-	Dialysate clear	-	Probable
Nicotera 2018 [33]	Italy	Case report	1	Lercanidipine	20 mg	53 y	-	150	-	2 y	-	Dialysate clear immediately	-	Probable
Gupta 2016 [13]	India	Case report	1	Amlodipine	5 mg	65 y	M	293.8	88.4	8 d	3 d	Dialysate clear within 24 h	Not-rechallenged	Probable
Moreiras 2014 [32]	Spain	Case report	1	Lercanidipine	5 mg	59 y	F	20	182	-	3 d	Dialysate clear within 24 h	Cloudy again	Certain (or definite)
Mallett 2012 [34]	Northern Ireland	Case report	1	Amlodipine	0.6 mg/kg	7 mo	M	57.5	164.6	5 mo	2 d	Dialysate clear before withdrawal ^b^	TG slightly increased in dialysate	Probable
Ram 2012 [31]	India	Case report	1	Diltiazem	-	55y	M	61	134	-	-	Dialysate clear after 1 d	Cloudy again	Certain (or definite)
Lopez 2011 [12]	Spain	Case report	1	Manidipine (from nifedipine OROS)	-	44 y	F	119	76	-	1 d	Dialysate clear within 24 h	-	Probable
Tsao 2009 [30]	Taiwan	Case report	1	Lercanidipine	10 mg	41 y	F	251	-	2 wk	3 d	Dialysate clear within 24 h	Cloudy again	Certain (or definite)
Roh 1999 [29]	Korea	Case report	1	Manidipine	40 mg	47 y	M	28	111	9 d	1 d	Dialysate clear after 1 d	Not-rechallenged	Probable
Tsurusaki 1995 [28]	Japan	Case report	1	Manidipine	20 mg	36 y	M	27.2	-	32 mo	4 d	Dialysate clear within 24 h	-	Probable
Fujii 1995 [27]	Japan	Case report	1	Manidipine	10 mg	58 y	M	26	-	2 mo	3 d	Dialysate clear within 24 h	-	Probable
Kato 1994 [26]	Japan	Case report	1	Manidipine	40 mg (From 20 mg)	51 y	F	29	121	4 mo	1 d	Dialysate clear within 12 h	-	Probable
Atsuko 1993 [25]	Japan	Case report	1	Manidipine	20 mg	44 y	M	24	81	6 mo	8 h	Dialysate clear within 1 d	Cloudy again	Certain (or definite)

^a^ Causality was assessed by the World Health Organization-Uppsala Monitoring Centre criteria and the Naranjo scale and subdivided into four categories: certain/definite, probable, possible and unlikely/doubtful. ^b^ The dialysate visibly cleared up, and triglycerides level in the dialysate normalised before withdrawal of the suspected CCB because dietary management of chyloperitoneum was initiated. Abbreviation: CP, chyloperitoneum; d, day(s); F, female; M, male; mo, months; OROS, osmotic-controlled release oral delivery system; PD, peritoneal dialysis; TGs, triglycerides; wk, weeks; y, years.

**Table 3:** Quality assessment of four cohort studies using a modified Newcastle–Ottawa Scale.

Study	Criteria of modified Newcastle–Ottawa Scale						
	Prior Criteria; Representativeness	Ascertainment of Exposure	Starting Condition Prior to Outcome	Adjustment for Confounding Factors ^a^	Ascertainment of Outcome	Sufficiency of Follow-Up Period	Adequacy of Follow-Up
Hsiao 2010 [21]	Lack of detailed description of selection criteria	Medical records	Chyloperitoneum was not pre sent	Infection and other causes	Standardised assessment	No statement	Complete follow-up
Yang 2008 [23]	Lack of detailed description of selection criteria	Medical records	Chyloperitoneum was not present	Infection and other causes	Standardised assessment	Follow-up period ≥ 30 d	More than 90%
Topal 2006 [22]	Unclear	Medical records	Chyloperitoneum was not present	Infection	Visual observation; Turbidity of peritoneal dialysate	Not sufficient; 1 d	Complete follow-up
Yoshimoto 1998 [11]	Participant selection by researchers	Medical records	Chyloperitoneum was not present	Infection and other causes	Standardised assessment	Not sufficient; 2 d	Subsequent observation by a biochemical test was performed on 53% of the patients

^a^ The factors except for infection were possible causes of chyloperitoneum, e.g., cancer, lymphatic obstruction, and traumatic abdominal injury. Abbreviation: d, day(s).

## Supplementary Materials

The following are available online at https://www.mdpi.com/1660-4601/16/8/1333/s1; Table S1: Checklist for Preferred Reporting Items for Systematic Reviews and Meta-Analyses; Table S2: Search strategy; Table S3: Criteria for modified Newcastle–Ottawa Scale; Figure S1: Forest plot showing the relationships between lercanidipine-associated chyloperitoneum and sex, with women as the reference group; and Figure S2: Forest plot showing age, duration of PD treatment, and serum triglyceride concentrations (standardised mean difference and 95% confidence interval) in the lercanidipine-associated chyloperitoneum group compared with those in the nonchyloperitoneum group.
