# Supplementary material for: Calcium Channel Blocker-Associated Chyloperitoneum in Patients Receiving Peritoneal Dialysis: A Systematic Review
Source: Int J Environ Res Public Health. 2019 Apr 13;16(8):1333. doi: 10.3390/ijerph16081333 (PMC6517940; doi:10.3390/ijerph16081333)
Supplement: Supplementary file 1 [file ijerph-16-01333-s001.pdf]

Table 1. Checklist for Preferred Reporting Items for Systematic Reviews and Meta-Analyses.

Section/topic	#	Checklist item	Reported on page #
**TITLE**			
Title	1	Identify the report as a systematic review, meta-analysis, or both.	1
**ABSTRACT**			
Structured summary	2	Provide a structured summary including, as applicable: background; objectives; data sources; study eligibility criteria, participants, and interventions; study appraisal and synthesis methods; results; limitations; conclusions and implications of key findings; systematic review registration number.	1
**INTRODUCTION**			
Rationale	3	Describe the rationale for the review in the context of what is already known.	1-2
Objectives	4	Provide an explicit statement of questions being addressed with reference to participants, interventions, comparisons, outcomes, and study design (PICOS).	2
**METHODS**			
Protocol and registration	5	Indicate if a review protocol exists, if and where it can be accessed (e.g., Web address), and, if available, provide registration information including registration number.	-
Eligibility criteria	6	Specify study characteristics (e.g., PICOS, length of follow-up) and report characteristics (e.g., years considered, language, publication status) used as criteria for eligibility, giving rationale.	2
Information sources	7	Describe all information sources (e.g., databases with dates of coverage, contact with study authors to identify additional studies) in the search and date last searched.	2
Search	8	Present full electronic search strategy for at least one database, including any limits used, such that it could be repeated.	2
Study selection	9	State the process for selecting studies (i.e., screening, eligibility, included in systematic review, and, if applicable, included in the meta-analysis).	2-3
Data collection process	10	Describe method of data extraction from reports (e.g., piloted forms, independently, in duplicate) and any processes for obtaining and confirming data from investigators.	3
Data items	11	List and define all variables for which data were sought (e.g., PICOS, funding sources) and any assumptions and simplifications made.	3
Risk of bias in individual studies	12	Describe methods used for assessing risk of bias of individual studies (including specification of whether this was done at the study or outcome level), and how this information is to be used in any data synthesis.	3
Summary measures	13	State the principal summary measures (e.g., risk ratio, difference in means).	3
Synthesis of results	14	Describe the methods of handling data and combining results of studies, if done, including measures of consistency (e.g., I^2) for each meta-analysis.	3
Risk of bias across studies	15	Specify any assessment of risk of bias that may affect the cumulative evidence (e.g., publication bias, selective reporting within studies).	-
Additional analyses	16	Describe methods of additional analyses (e.g., sensitivity or subgroup analyses, meta-regression), if done, indicating which were pre-specified.	-
**RESULTS**			
Study selection	17	Give numbers of studies screened, assessed for eligibility, and included in the review, with reasons for exclusions at each stage, ideally with a flow diagram.	3-4

Study characteristics	18	For each study, present characteristics for which data were extracted (e.g., study size, PICOS, follow-up period) and provide the citations.	4
Risk of bias within studies	19	Present data on risk of bias of each study and, if available, any outcome level assessment (see item 12).	8-9
Results of individual studies	20	For all outcomes considered (benefits or harms), present, for each study: (a) simple summary data for each intervention group (b) effect estimates and confidence intervals, ideally with a forest plot.	5-7
Synthesis of results	21	Present results of each meta-analysis done, including confidence intervals and measures of consistency.	6
Risk of bias across studies	22	Present results of any assessment of risk of bias across studies (see Item 15).	6
Additional analysis	23	Give results of additional analyses, if done (e.g., sensitivity or subgroup analyses, meta-regression [see Item 16]).	-
**DISCUSSION**			
Summary of evidence	24	Summarize the main findings including the strength of evidence for each main outcome; consider their relevance to key groups (e.g., healthcare providers, users, and policy makers).	10-11
Limitations	25	Discuss limitations at study and outcome level (e.g., risk of bias), and at review-level (e.g., incomplete retrieval of identified research, reporting bias).	11
Conclusions	26	Provide a general interpretation of the results in the context of other evidence, and implications for future research.	11
**FUNDING**			
Funding	27	Describe sources of funding for the systematic review and other support (e.g., supply of data); role of funders for the systematic review.	12

Table 2. Search strategy.

**PubMed**	
#1	calcium channel blocker OR calcium channel blockers[MeSH terms]
#2	lacidipine OR nilvadipine OR manidipine OR barnidipine OR lercanidipine OR cilnidipine OR benidipine OR amlodipine OR felodipine OR isradipine OR nicardipine OR nisoldipine OR nimodipine OR nifedipine OR verapamil OR diltiazem
#3	((cloudy OR turbid) and (ascitic fluid OR peritoneal fluid OR dialysate)) OR chylous ascites[MeSH terms] OR chyloperitoneum OR chylous ascites
#4	adverse drug reaction[MeSH terms] OR adverse drug event OR adverse event OR adverse effect OR side effect OR drug toxicity[MeSH Terms] OR safety[MeSH terms]
#5	peritoneal dialysis[MeSH terms] OR peritoneal dialysis
#6	#1 AND #3
#7	#1 AND #5
#8	#2 AND #3
#9	#2 AND #5
**EMBASE**	
#1	'calcium channel blocking agent'
#2	'peritoneal dialysis'
#3	safety OR toxicity OR 'adverse drug reaction' OR 'side effect'
#4	#1 AND #2
#5	'chlyous ascites' OR chyloperitoneum
#6	(turbid OR cloudy) AND ('peritoneal fluid' OR 'ascites fluid' OR dialysate)
#7	#1 AND #5
#8	#1 AND #6
#9	lacidipine OR nilvadipine OR manidipine OR barnidipine OR lercanidipine OR cilnidipine OR benidipine OR amlodipine OR felodipine OR isradipine OR nicardipine OR nisoldipine OR nimodipine OR nifedipine OR verapamil OR diltiazem
#10	#2 AND #3 AND #9
#11	#5 AND #9
#12	#6 AND #9
**Cochrane**	
#1	Calcium channel blocker AND peritoneal dialysis
#2	Calcium channel blocker AND chylous ascites
#3	Calcium channel blocker AND chyloperitoneum
#4	Calcium channel blocker AND (turbid peritoneal OR cloudy peritoneal)
#5	(lacidipine OR nilvadipine OR manidipine OR barnidipine OR lercanidipine OR cilnidipine OR benidipine OR amlodipine OR felodipine OR isradipine OR nicardipine OR nisoldipine OR nimodipine OR nifedipine OR verapamil OR diltiazem) AND peritoneal dialysis
#6	(lacidipine OR nilvadipine OR manidipine OR barnidipine OR lercanidipine OR cilnidipine OR benidipine OR amlodipine OR felodipine OR isradipine OR nicardipine OR nisoldipine OR nimodipine OR nifedipine OR verapamil OR diltiazem) AND chylous ascites
#7	(lacidipine OR nilvadipine OR manidipine OR barnidipine OR lercanidipine OR cilnidipine OR benidipine OR amlodipine OR felodipine OR isradipine OR nicardipine OR nisoldipine OR nimodipine OR nifedipine OR verapamil OR diltiazem) AND chyloperitoneum
#8	(lacidipine OR nilvadipine OR manidipine OR barnidipine OR lercanidipine OR cilnidipine OR benidipine OR amlodipine OR felodipine OR isradipine OR nicardipine OR nisoldipine OR nimodipine OR nifedipine OR verapamil OR diltiazem) AND (turbid peritoneal OR cloudy peritoneal)
**RISS**	
#1	(calcium channel blocker) (chyloperitoneum | chylous ascites | turbid peritoneal | cloudy peritoneal)
#2	(calcium antagonist) (chyloperitoneum | chylous ascites | turbid peritoneal | cloudy peritoneal)
#3	(peritoneal dialysis) (calcium channel blocker | calcium antagonist)
#4	(lacidipine | nilvadipine | manidipine | barnidipine | lercanidipine | cilnidipine | benidipine | amlodipine |

	felodipine | isradipine | nicardipine | nisoldipine | nimodipine | nifedipine | verapamil | diltiazem) (chyloperitoneum | chylous ascites | turbid peritoneal | cloudy peritoneal)
#5	(lacidipine | nilvadipine | manidipine | barnidipine | lercanidipine | cilnidipine | benidipine | amlodipine | felodipine | isradipine | nicardipine | nisoldipine | nimodipine | nifedipine | verapamil | diltiazem) (peritoneal dialysis)
**CiNii**	
#1	calcium channel blocker AND chyloperitoneum
#2	calcium channel blocker AND chylous ascites
#3	calcium channel blocker AND turbid peritoneal
#4	calcium channel blocker AND cloudy peritoneal
#5	calcium channel blocker AND peritoneal dialysis
#6	calcium antagonist AND chyloperitoneum
#7	calcium antagonist AND chylous ascites
#8	calcium antagonist AND turbid peritoneal
#9	calcium antagonist AND cloudy peritoneal
#10	calcium antagonist AND peritoneal dialysis
#11	lacidipine AND (chyloperitoneum OR chylous ascites OR turbid peritoneal OR cloudy peritoneal)
#12	nilvadipine AND (chyloperitoneum OR chylous ascites OR turbid peritoneal OR cloudy peritoneal)
#13	manidipine AND chyloperitoneum
#14	manidipine AND chylous ascites
#15	manidpine AND turbid peritoneal
#16	manidipine AND cloudy peritoneal
#17	barnidipine AND (chyloperitoneum OR chylous ascites OR turbid peritoneal OR cloudy peritoneal)
#18	lercanidipine AND (chyloperitoneum OR chylous ascites OR turbid peritoneal OR cloudy peritoneal)
#19	cilnidipine AND (chyloperitoneum OR chylous ascites OR turbid peritoneal OR cloudy peritoneal)
#20	benidipine AND (chyloperitoneum OR chylous ascites OR turbid peritoneal OR cloudy peritoneal)
#21	amlodipine AND chylous ascites
#22	amlodipine AND (chyloperitoneum AND turbid peritoneal AND cloudy peritoneal)
#23	felodipine AND (chyloperitoneum OR chylous ascites OR turbid peritoneal OR cloudy peritoneal)
#24	isradipine AND (chyloperitoneum OR chylous ascites OR turbid peritoneal OR cloudy peritoneal)
#25	nicardipine AND (chyloperitoneum OR chylous ascites OR turbid peritoneal OR cloudy peritoneal)
#26	nisoldipine AND (chyloperitoneum OR chylous ascites OR turbid peritoneal OR cloudy peritoneal)
#27	nimodipine AND (chyloperitoneum OR chylous ascites OR turbid peritoneal OR cloudy peritoneal)
#28	nifedipine AND (chyloperitoneum OR chylous ascites OR turbid peritoneal OR cloudy peritoneal)
#29	verapamil AND (chyloperitoneum OR chylous ascites OR turbid peritoneal OR cloudy peritoneal)
#30	diltiazem AND (chyloperitoneum OR chylous ascites OR turbid peritoneal OR cloudy peritoneal)

Table 3. Criteria for modified Newcastle-Ottawa Scale.

Item	Criteria	Score
**SELECTION**		
1. Representativeness of the exposed cohort	Population-based study, random recruitment of participants, or consecutive enrollment of participants	One point
	Participant selection by researchers or voluntary participants	Zero point
	Unclear method of participant selection	Zero point
2. Ascertainment of exposure	Medical record or structured interview	One point
	Self-report	Zero point
	No statement or unclear method of exposure ascertainment	Zero point
3. Demonstration that chyloperitoneum was not present at start of study	Yes	One point
	No	Zero point
**COMPARABILITY**		
1. Comparability of cohorts on the basis of the design or analysis	Adjustment or exclusion of the confounding factors for chyloperitoneum	One point
	No adjustment or control of the confounding factors for chyloperitoneum	Zero point
	No statement	Zero point
**OUTCOME**		
1. Ascertainment of outcome	Standardized assessment or confirmation of chyloperitoneum in the medical record	One point
	Self-report or visual observation of turbidity	Zero point
	No statement	Zero point
2. Enough period of follow-up for chyloperitoneum to occur	Yes	One point
	No	Zero point
	No statement	Zero point
3. Adequacy of follow-up of cohorts	Complete follow up of more than 90% of enrolled participants	One point
	More than 10% of subjects lost to follow up	Zero point
	No statement or unknown due to unclear method of study	Zero point

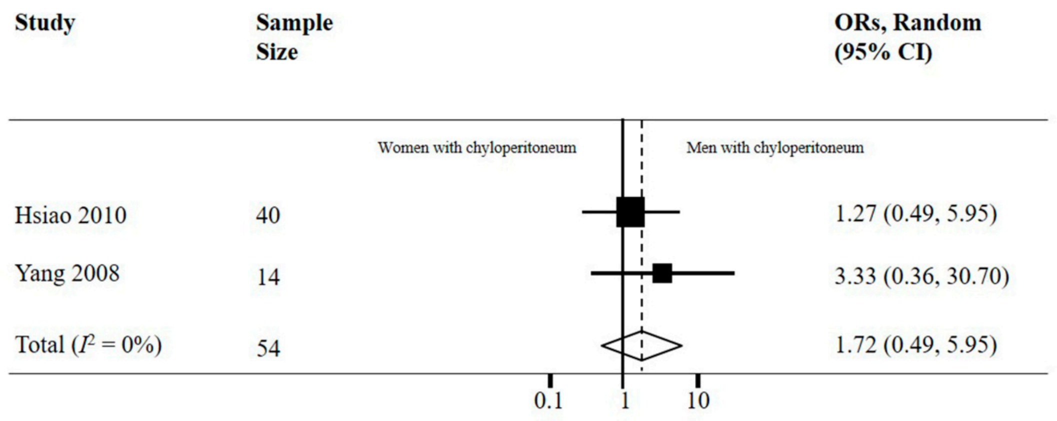

Figure 1. Forest plot showing the relationships between lercanidipine-associated chyloperitoneum and sex, with women as the reference group. Abbreviation: CI, confidence interval; OR, odds ratio.

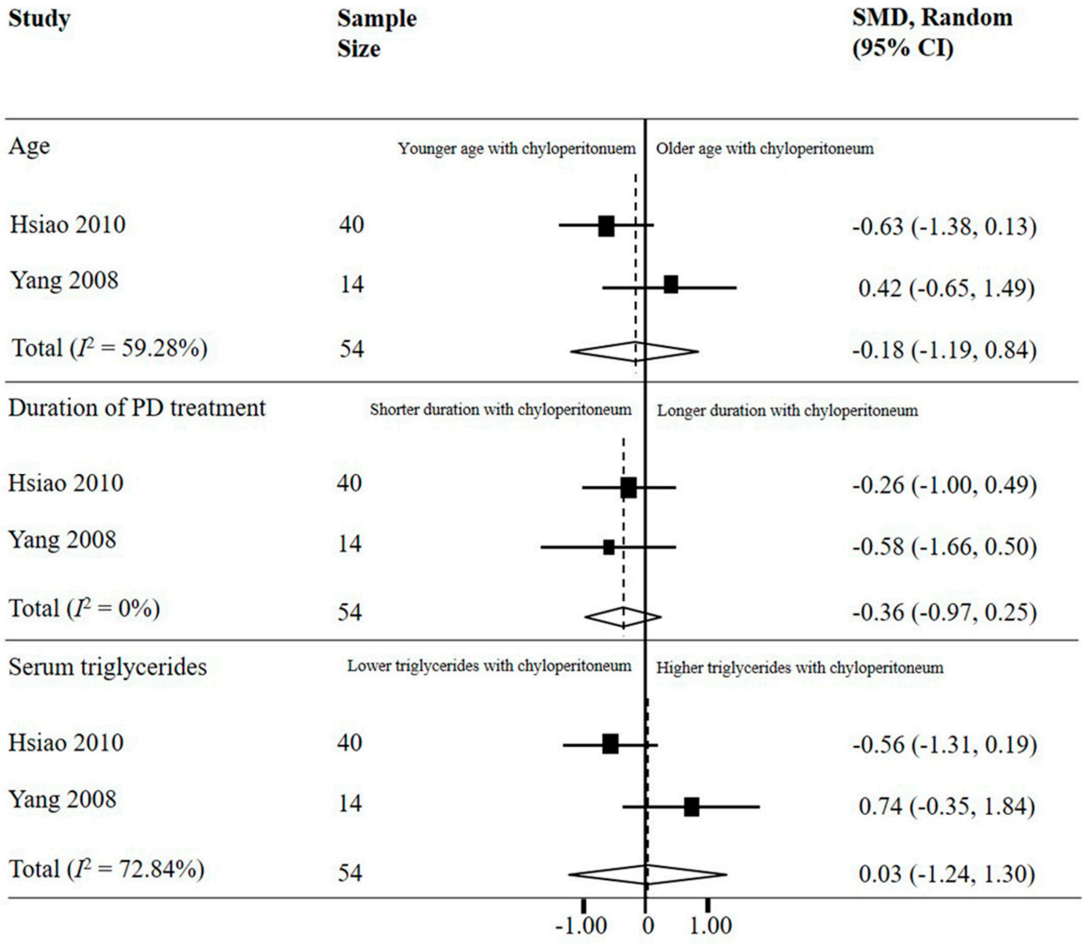

Figure 2. Forest plot showing age, duration of PD treatment, and serum triglyceride concentrations (standardised mean difference and 95% confidence interval) in the lercanidipine-associated chyloperitoneum group compared with those in the nonchyloperitoneum group. Abbreviation: CI, confidence interval; PD, peritoneal dialysis; SMD, standardised mean difference.
